# First Report of Small Skeletal Fossils from the Upper Guojiaba Formation (Series 2, Cambrian), Southern Shaanxi, South China

**DOI:** 10.3390/biology12070902

**Published:** 2023-06-24

**Authors:** Mei Luo, Fan Liu, Yue Liang, Luke C. Strotz, Jiayue Wang, Yazhou Hu, Baopeng Song, Lars E. Holmer, Zhifei Zhang

**Affiliations:** 1 State Key Laboratory of Continental Dynamics, Shaanxi Key Laboratory of Early Life & Environments, Department of Geology, Northwest University, Xi’an 710069, China; meiluo_nwu@outlook.com (M.L.); liufan@nwu.edu.cn (F.L.); yueliang_nwu@outlook.com (Y.L.); lukestrotz@nwu.edu.cn (L.C.S.); wangjiayue@stumail.nwu.edu.cn (J.W.); huyz_nwu@163.com (Y.H.); baopeng_nwu@stumail.nwu.edu.cn (B.S.); lars.holmer@pal.uu.se (L.E.H.); 2 Biodiversity Institute and Department of Ecology & Evolutionary Biology, University of Kansas, Lawrence, KS 66045, USA; 3 Department of Palaeontology, University of Vienna, Althanstrasse 14, 1090 Vienna, Austria; 4 Palaeobiology, Department of Earth Sciences, Uppsala University, SE-752 36 Uppsala, Sweden

**Keywords:** small skeletal fossils (SSFs), fossil assemblages, early Cambrian, biostratigraphy, southern Shaanxi

## Abstract

**Simple Summary:**

Small skeletal fossils are reported for the first time from the early Cambrian Guojiaba Formation, southern Shaanxi, China. All specimens were recovered from bioclastic limestone interbeds and encompass a wide variety of skeletal clades, including brachiopods, sphenothallids, archaeocyaths, bradoriids, sponge spicules, echinoderm plates, and trilobite spines. The archaeocyaths described herein are considerably older than those described from the Xiannvdong Formation, which was previously assumed to contain the lowest archaeocyath-bearing horizons in South China. The brachiopod *Lingulellotreta yuanshanensis* is recorded for the first time from the Fucheng area, with previous records confined mainly to the Chengjiang Fauna-bearing Yu’anshan Formation in the lower Cambrian, the eastern Yunnan Province. Micro-morphological and elemental analyses of the small skeletal fossil assemblages were carried out using SEM, BSEM, and Micro X–ray fluorescence. The skeletal fauna in the Guojiaba Formation resembles the assemblages recovered from the upper Yu’anshan Formation (Chengjiang Fauna) in eastern Yunnan Province. Based on the recovered assemblage, the biostratigraphic age of the Guojiaba Formation correlates with the Chiungchussuan Stage (Stage 3 of Cambrian Series 2).

**Abstract:**

A small skeletal fossil assemblage is described for the first time from the bioclastic limestone interbeds of the siltstone-dominated Guojiaba Formation, southern Shaanxi, China. The carbonate-hosted fossils include brachiopods (*Eohadrotreta zhujiahensis*, *Eohadrotreta zhenbaensis*, *Spinobolus* sp., *Kuangshanotreta malungensis*, *Kyrshabaktella* sp., *Lingulellotreta yuanshanensis*, *Eoobolus incipiens*, and *Eoobolus* sp.), sphenothallids (*Sphenothallus* sp.), archaeocyaths (*Robustocyathus* sp. and *Yukonocyathus* sp.), bradoriids (*Kunmingella douvillei*), chancelloriids sclerites (*Onychia* sp., *Allonnia* sp., *Diminia* sp., *Archiasterella pentactina*, and *Chancelloria* cf. *eros*), echinoderm plates, fragments of trilobites (*Eoredlichia* sp.), and hyolithelminths. The discovery of archaeocyaths in the Guojiaba Formation significantly extends their stratigraphic range in South China from the early Tsanglangpuian at least to the late Chiungchussuan. Thus, the Guojiaba Formation now represents the lowest known stratigraphic horizon where archaeocyath fossils have been found in the southern Shaanxi area. The overall assemblage is most comparable, in terms of composition, to Small skeletal fossil (SSF) assemblages from the early Cambrian Chengjiang fauna recovered from the Yu’anshan Formation in eastern Yunnan Province. The existing position that the Guojiaba Formation is correlated with Stage 3 in Cambrian Series 2 is strongly upheld based on the fossil assemblage recovered in this study.

## 1. Introduction

Small skeletal fossils (SSFs) are one of the primary index fossils in Cambrian sequences and are usually preserved as phosphatized fragments of shells, valves, and various tubular steinkerns [1,2,3,4,5,6,7,8,9,10]. SSFs include a wide variety of invertebrate clades, such as brachiopods, mollusks, sponges, and various other organisms [11,12,13,14,15,16]. Typically millimeters in size or smaller, the original morphology and microstructure of SSFs are often well-preserved, making them ideal for studying the early evolution of mineralized metazoans [17,18,19]. SSF assemblages can provide important information about past ecosystems and facilitate palaeoclimatic and palaeoenvironmental reconstructions [20,21,22,23].

Cambrian Series 2 Stage 3 depositional sequences are widely exposed and well-developed in the region of the South China Platform, including in southern Shaanxi Province, western Hubei Province, western Sichuan Province, and eastern Yunnan Province [7,9,10,11,24,25,26,27]. The Xihaoping Member of Dengying Formation, Shuijingtuo Formation, Guojiaba Formation, Yu’anshan Formation, and the upper Jiulaodong Formation contain abundant SSFs in the South China Platform [9,22,24,27]. Hyolithelminths, hyoliths, echinoderm plates, archaeocyaths, bradoriids, chancelloriids, sphenothallids, and brachiopods, are all commonly found in the above formations [11,28,29,30]. Because benthic trilobites, which are highly regional and difficult to correlate stratigraphically on a large scale [6,31,32], are generally endemic and conspecies-absent from these formations, these SSF assemblages across such deposition sequences have become an important biostratigraphic tool for dating, subdividing and correlating the early Cambrian deposits of South China [33].

For the first time, we systematically describe the SSF assemblage from the bioclastic limestone interbeds of the Cambrian Series 2, Stage 3 Guojiaba Formation. The fauna comprises three species of acrotretid brachiopods, four species of lingulid brachiopods, one species of sphenothallid, and two species of archaeocyaths. The discovery of the archaeocyaths *Robustocyathus* sp. and *Yukonocyathus* sp. in the Guojiaba Formation at the Dayingcun Section makes the Guojiaba Formation the lowest-known stratigraphic level where archaeocyaths occur in the Fucheng area. This SSF assemblage both expands knowledge of the overall Guojiaba fossil fauna, which has previously only been reconstructed using macrofossil material collected from shales and siltstones, and enhances stratigraphic correlation across South China.

## 2. Geological Background

**Figure 1:**
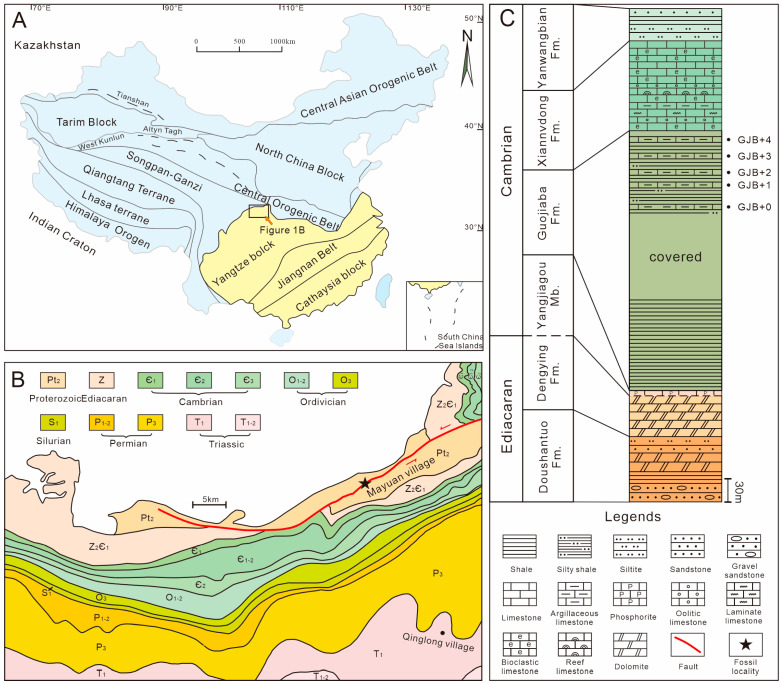
Geological map, sample locality, and the early Cambrian stratigraphy of the Fucheng area, southern Shaanxi, South China. (**A**) Geological map showing the distribution of China’s major continental blocks, terranes, and suture belts; the yellow represents the South China Platform. (**B**) Regional geological map showing the distribution of Cambrian strata in the Fucheng area and the location (marked by a black five-pointed star) of the section investigated and sampled (inset) from. (**C**) Stratigraphic column of the Dayingcun Section, note the levels at which carbonate samples were collected for acid maceration.

China consists of tectonically stable platforms such as the South China, North China, Qiadam, and Tarim platforms, along with some orogenic belts and terranes (Figure 1A). The well-developed and continuous Cambrian strata found across these platforms provide extensive material for studying early metazoans [7,9,10,34,35,36,37,38]. The Dayingcun Section is located near Micang Mountain in Fucheng Town, Nanzheng County, Shaanxi Province, on the northwest margin of the South China Platform (Figure 1B). Upper Ediacaran and lower Cambrian deposits are widely distributed in the Fucheng area, with deposits assigned to the Dengying, Guojiaba, Xiannvdong, and Yanwangbian Formations in ascending order (Figure 1C). The Guojiaba Formation is fairly consistent in the Micang and Daba Mountains, with essentially the same lithology but increasing in thickness towards the west, reaching over 500 m in the Nanzheng, Ningqiang, and Nanjiang areas [39]. The Guojiaba Formation has a thickness of 358 m in the Dayingcun Section. The lithology consists of grey mudstone, siltstone, and black shale, with several thin interbedded bioclastic limestone beds, and is conformably overlain by the Xiannvdong Formation, although it is disconformably overlain by the Dengying Formation in some areas. The Guojiaba Formation is divided into three parts based on lithological changes. The lower part of the Guojiaba Formation consists mainly of green-yellow shale. The middle part of the Guojiaba Formation consists of grey-black calcareous shale. The upper part of the Guojiaba Formation consists mainly of grey-black calcareous shale interbedded by bioclastic limestone.

## 3. Materials and Methods

All microfossils in this study came from the upper part of the Guojiaba Formation in the Dayingcun Section, Fucheng area, southern Shaanxi. The original samples of 160 kg were crushed into 7–15 cm diameter particles before being placed in 7–10% acetic acid solution. The insoluble residue was cleaned and dried after approximately three days. Fragments were manually picked using a binocular microscope. SEM images of gold-coated fossils were taken using a Phenom XL G2 SEM with a resolution of 8 kV and 0.10 Pa. Thin sections of all sampled layers were prepared at Northwest University. Micro X-ray fluorescence was performed on thin sections to assess elemental composition. The similarity of the SSF fauna in the Guojiaba formation to other SSF faunas was undertaken using multivariate cluster analysis (based on Jaccard similarity), performed using PAST. All fossil specimens from this study are housed in the collections of the Northwest University Early Life Institute (ELI), Xi’an, China.

## 4. Small Skeletal Fossils and Elemental Analysis of the Guojiaba Formation

Abundant and diverse SSFs were recovered from the bioclastic limestone at the top of the Cambrian Guojiaba Formation. The skeletal fossils are mainly preserved by phosphatization. In terms of faunal composition, the SSF fauna of the Dayingcun Section includes brachiopods, sphenothallids, archaeocyaths, chancelloriids, hyolithelminths, oocysts, echinoderms, librigena (trilobites), and bradoriids.

**Figure 2:**
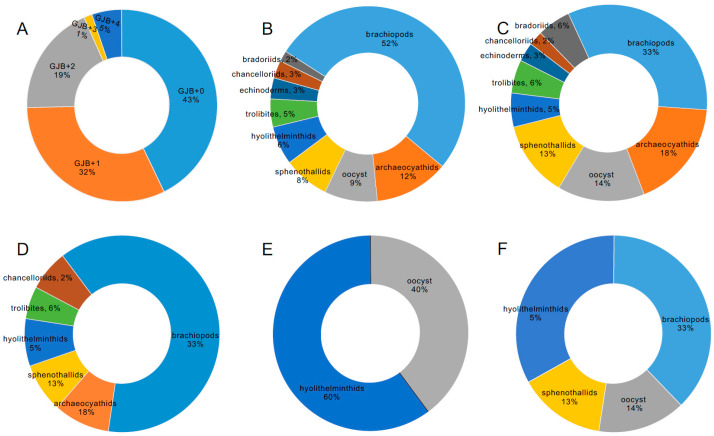
Comparisons of relative abundance for samples from the Guojiaba Formation. (**A**) Relative abundance of specimens for each sample. (**B**) Relative abundances for GJB+0. (**C**) Relative abundances for GJB+1. (**D**) Relative abundances for GJB+2. (**E**) Relative abundances for GJB+3. (**F**) Relative abundances for GJB+4.

All 451 SSF specimens were recovered from Guojiaba Formation residues. GJB+0 accounted for 43%, GJB+1 for 32%, GJB+2 for 19%, GJB+3 for 1%, and GJB+4 for 5% of the total abundance (Figure 2A). A total of 171 SSFs were recovered from sample site GJB+0. Brachiopods represented 52%, archaeocyaths 12%, oocysts 9%, sphenothallids 8%, hyolithelminths 6%, trilobites 5%, echinoderms 3%, chancelloriids 3%, and bradoriids 2% (Figure 2B). A total of 127 SSFs were recovered from sample site GJB+1, of which 33% were brachiopods, 18% archaeocyaths, 14% oocysts, 13% sphenothallids, 5% hyolithelminths, 6% trilobites, 3% echinoderms, 2% chancelloriids, and 6% bradoriids (Figure 2C). At sample site GJB+2, a total of 132 SSFs were recovered, of which 33% were brachiopods, 18% archaeocyaths, 13% sphenothallids, 5% hyolithelminths, 6% trilobites, and 2% chancelloriids (Figure 2D). At sample site GJB+3, only five SSFs were found; three of hyolithelminths and two of oocysts (Figure 2E). At sample site GJB+4, 21 SSFs were presented, of which 33% were brachiopods, 14% oocysts, 13% sphenothallids, and 5% hyolithelminths (Figure 2F). 

**Figure 3:**
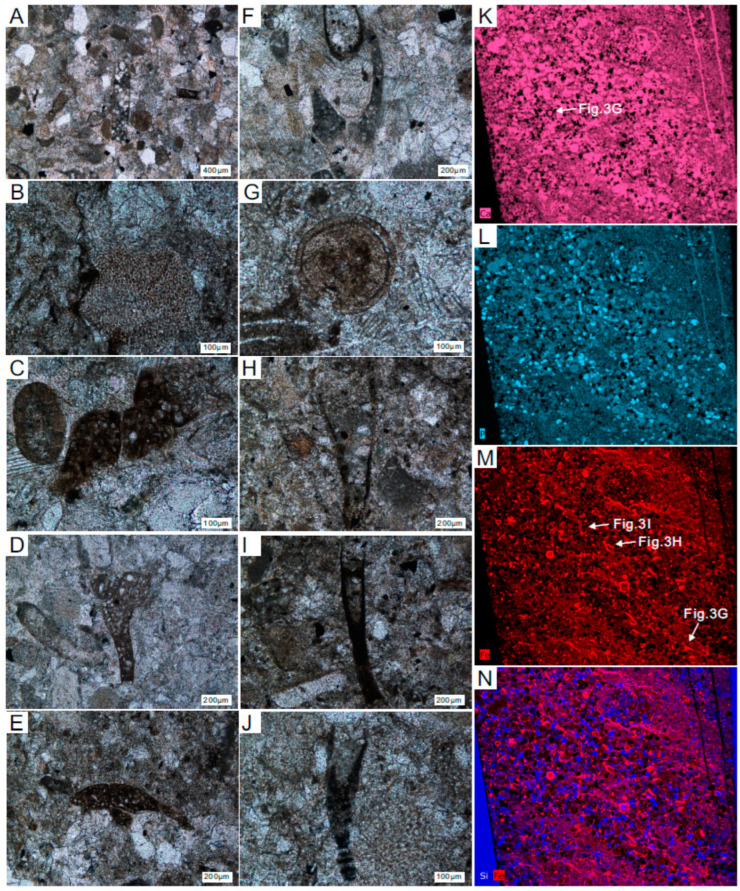
Thin section microphotographs and elemental maps from sample site GJB+0 (**A**–**J**). SSFs were discovered from the thin section of the sample site GJB+0. (**A**) Partial view; (**B**) indeterminate algae; (**C**) *Onychia* sp.; (**D**) *Allonnia* sp.; (**E**,**F**) *Diminia* sp.; (**G**) Oocyst; (**H**) hyolithelminth; (**I**,**J**) a cross-section of *Sphenothallus* sp.; (**K**–**N**) different element distribution images in the thin section; (**K**) Ca = calcium; (**L**) P = phosphorus; (**M**) Fe = iron; (**N**) Si and Fe = silicon and iron.

Thin section results for the four sample sites are consistent across all four samples. We selected the thin section for GJB+0 as the best representative of all sample sites because this thin section is the most distinct and clear. Some skeletal fossils can be identified in the GJB+0 thin section, including hyolithelminths (Figure 3A,H), indeterminate algae (Figure 3B), chancelloriid spicules of *Onychia* sp. (Figure 3C), *Allonnia* sp. (Figure 3D), *Diminia* sp. (Figure 3E,F), oocysts (Figure 3G), and sphenothallids of *Sphenothallus* sp. (Figure 3I,J). Results of μ-XRF scanning and analysis of the thin section from GJB+0 indicate that the relatively abundant elements include calcium (=Ca) (Figure 3K), phosphorus (=P) (Figure 3L), iron (=Fe) (Figure 3M), and silicon (=Si) (Figure 3N). The main elemental components of the fossils are calcium phosphate (Figure 3K,L) and iron, with the iron coating the shell surfaces (Figure 3M).

**Figure 4:**
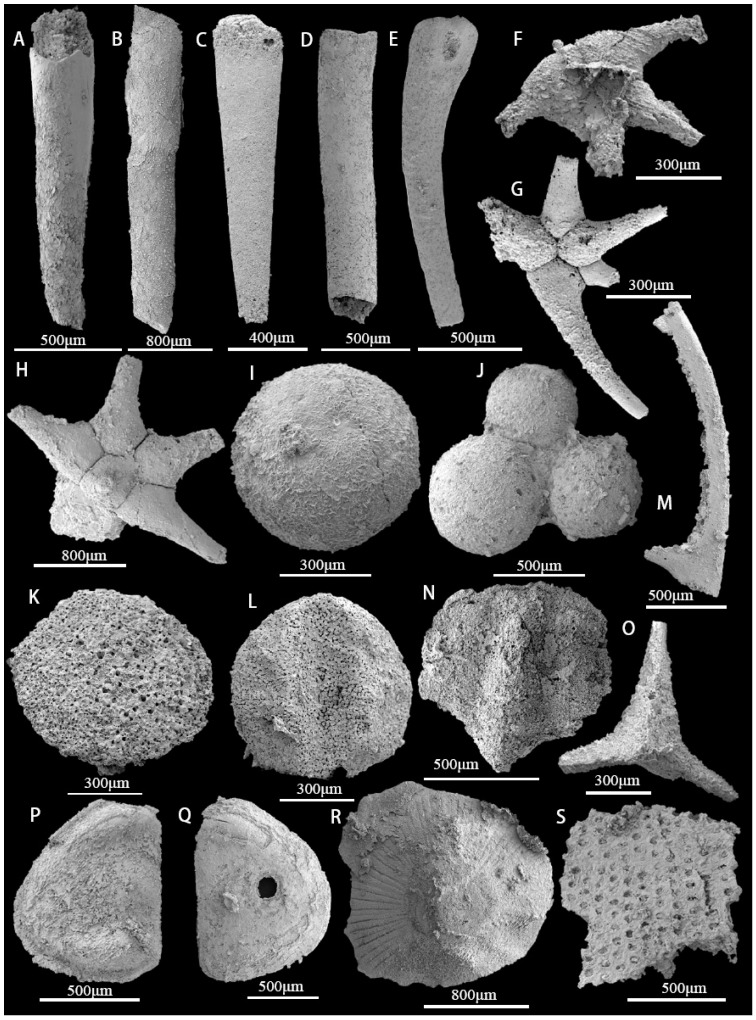
SSFs from the bioclastic limestone interbeds of the upper Guojiaba Formation. (**A**–**D**) *Hyolithellus* sp., ELI–DYC–GJB+0-22, ELI–DYC–GJB+1–31, ELI–DYC–GJB+2–17, ELI–DYC–GJB+4–15, and internal molds; (**E**) *Chancelloria* sp., ELI–DYC–GJB+1–34; (**F**) *Onychia* sp., ELI–DYC–GJB+0–69; (**G**) *Archiasterella pentactina*, ELI–DYC–GJB+0–106; (**H**) *Chancelloria* cf. *eros*, ELI–DYC–GJB+0–102; (**I**) an isolated oocyst, ELI–DYC–GJB+1–28; (**J**) Multiple oocyst, ELI–DYC–GJB+1–12; (**K**,**L**) Echinoderm plates, ELI–DYC–GJB+0–41, ELI–DYC–GJB+0–23; left librigena of trilobite, ELI–DYC–GJB+1–11; (**M**) left librigena of trilobite, ELI–DYC–GJB+1–11; (**N**) incomplete cranidium that may belong to the trilobite taxon *Eoredlichia* sp., ELI–DYC–GJB+0–29; (**O**) thoracic axial rings with the axial spine, ELI–DYC–GJB+0–107; (**P**,**Q**) *Kunmingella douvillei*, ELI–DYC–GJB+0–30, ELI–DYC–GJB+0–101, the latter with possible drill hole; (**R**) cap-like mollusc with radial ornamentation, ELI–DYC–GJB+0–90; (**S**) fragments of archaeocyath *Robustocyathellus* sp., ELI–DYC–GJB+1–3.5. Systematic Paleontology.

The majority of hyolithelminthids are preserved as phosphatic internal molds without detailed shell ornamentation or microstructure (Figure 4A–D). Chancelloriidae (*Chancelloria* sp., *Onychia* sp., *Archiasterella pentactina*, *Chancelloria* cf. *eros*) (Figure 4E–H), oocysts (Figure 4I,J), and echinoderm plates (Figure 4K,L) are all relatively rare. In addition to trilobites (Figure 4M–O), the arthropod assemblage includes a bradoriid carapace, identified as *Kunmingella douvillei* (Figure 4P,Q). There is also a cup-like mollusc of indeterminate affinity (Figure 4R). The remaining material consists of a large number of archaeocyath fragments that have undergone secondary phosphatization (Figure 4S). Brachiopods represent the most abundant and diverse component of the overall assemblage (Figure 2B–D,F). Seven brachiopod taxa are present, belonging to the orders Acrotretida and Lingulida (see Part 6 for further details) [40,41]. The most common taxa are the two species of acrotretoid brachiopods, *Eohadrotreta zhujiahensis* and *E. zhenbaensis* [13,24], which collectively account for 37.6% of the total number of brachiopods. The five remaining species, *Kyrshabaktella* sp., *Spinobolus* sp., *Lingulellotreta yuanshanensis*, *Eoobolus* aff. *viridis*, and *Eoobolus* sp. belong to the Lingulida. Most brachiopod valves are fragmented and incomplete; however, some juveniles have conjoined shells. Sphenothallids, including *Sphenothallus* sp. are the most abundant non-brachiopod element of the fauna and have a variety of skeletal forms. Some specimens have possible drill holes.

## 5. Systematic Paleontology

Subphylum Linguliformea Williams and Others, 1996

Class Lingulata Gorjansky Popov, 1985

Order Acrotretida Kuhn, 1949

Superfamily Acrotretoidea Schuchert, 1893

Family Acrotretidae Schuchert, 1893

Genus *Eohadrotreta* Li and Holmer, 2004

Type species: *Eohadrotreta zhujiahensis* Li and Holmer, 2004

*Eohadrotreta zhujiahensis* Li and Holmer, 2004

(Figure 5A–H)

2004 *Eohadrotreta zhujiahensis* Li and Holmer, pp. 206–208, Figures 14 and 15 in [24].

2017 *Eohadrotreta zhujiahensis* Zhang et al., Figures 7–10 in [12].

2018 *Eohadrotreta zhujiahensis* Zhang et al., Figure 4 in [8].

**Material.** Seventeen specimens with conjoined valves, eighteen specimens with ventral valves, and twelve specimens with dorsal valves from the Guojiaba Formation.

**Diagnosis.** See Zhang et al. [7].

**Figure 5:**
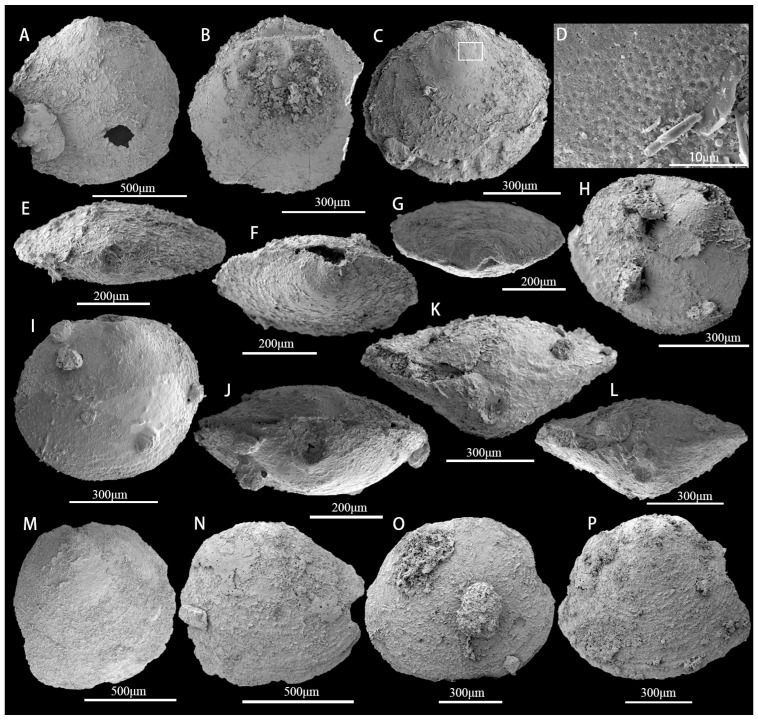
Acrotretida brachiopods from the upper Guojiaba Formation of the Dayingcun Section. (**A**–**H**) *Eohadrotreta zhujiahensis*, ELI–DYC–GJB+1–19, ELI–DYC–GJB+1–11, ELI–DYC–GJB+0–112, ELI–DYC–GJB+0–83, ELI–DYC–GJB+0–56, ELI–DYC–GJB+0–73, ELI–DYC–GJB+0–68, and ventral valve; (**A**,**C**) ventral view; (**B**) dorsal valve; (**D**) detailed view of pitting structure enlarged in (**C**); (**E**–**G**) posterior view; (**H**) oblique front view of ventral valve; (**I**–**L**) *Eohadrotreta zhenbaensis*, ELI–DYC–GJB+0–35, ELI–DYC–GJB+0–58, ELI–DYC–GJB+0–87; (**I**) external; (**J**) posterior; (**K**,**L**) posterior view of conjunct valves; (**M**–**P**) *Kuangshanotreta malungensis*, ELI–DYC–GJB+4–5, ELI–DYC–GJB+2–2, ELI–DYC–GJB+0–5, ELI–DYC–GJB+2–6, and ventral valve.

**Description.** The commissural contour is usually transversely oval, and the shell is biconvex or slightly ventribiconvex (Figure 5A–C). Growth lines are present in the later growth stages (Figure 5C). The lateral and posterior margins of the commissural contour are strongly arched, whereas the anterior margin is gently rounded (Figure 5C). Slender, flat-bottomed, hemispherical pits (approximately 1 μm in diameter) ornament the larval valve (Figure 5D). The ventral pseudointerarea is rudimentary or weakly developed. The external pedicle foramen is circular and not enclosed by the larval shell. The dorsal valve is slightly convex (Figure 5B). In the dorsal view, the dorsal larval valve has a circular or subcircular outline (Figure 5B), and the dorsal pseudointerarea is orthocline. The average length of the ventral valve is 632 μm but varies from 478 μm to 907 μm (Table 1). The ventral valve is cap-like (Figure 5H), on average 95% as long as wide. The metamorphic shell is elevated above the shell surface. The comparatively large pedicle foramen is approximately 76 μm long and 99 μm wide, with an average length/width ratio of 106% (Figure 5E–G). The tongue-like apical process encircles the pedicle foramen and occupies an average of 30% of the valve length. The ventral cardinal muscle scars are weakly developed and occupy approximately 22% of the valve length (Table 1).

**Remarks.** *Eohadrotreta zhenbaensis* is generally similar in shell outline to *E. zhujiahensis*, but there are substantial ontogenetic differences between the two species [8]. The pedicle foramen of *E. zhenbaensis* is never enclosed within the metamorphic shell, whereas, after the pedicle foramen enclosing stage, the pedicle foramen of *E. zhujiahensis* is enclosed within the metamorphic shell [8]. The ventral pseudointerarea of *E. zhenbaensis* is almost catacline to procline, divided by a narrow elongate intertrough, but the ventral pseudointerarea of *E. zhujiahensis* is almost catacline, divided by a slightly wider and short intertrough [8]. The ventral cardinal muscle scars are different for *E. zhenbaensis* versus *E. zhujiahensis,* with the former being more pronounced than the latter [8]. The dorsal pseudointerarea type of both are consistent between the two taxa and the dorsal cardinal muscle scars are well developed. *Eohadrotreta zhenbaensis* has a better-developed median septum than *E. zhujiahensis*. It has been previously proposed that *E. zhujiahensis* could represent a juvenile stage of *E. zhenbaensis* [13].

**Occurrence.** The upper part of the Guojiaba Formation (lower Cambrian, Chiungchussuan Stage) of southern Shaanxi Province; Shuijingtuo Formation of western Hubei Province, South China.

Type species: *Eohadrotreta zhenbaensis* Li and Holmer, 2004

*Eohadrotreta zhenbaensis* Li and Holmer, 2004

(Figure 5I–L)

2004 *Eohadrotreta zhenbaensis* Li and Holmer, pp. 206–208, Figures 11–13 in [24].

2007 *Eohadrotreta zhenbaensis* Holmer and Popov, pp. 2560–2562, Figures 1693 and 1694 in [41].

2015 *Eohadrotreta zhenbaensis* Yang et al., Figure 9E,F in [22].

2016 *Eohadrotreta zhenbaensis* Zhang et al., Figures 2–5 in [7].

2016 *Eohadrotreta zhenbaensis* He et al., Figures 4 and 5 in [42].

2017 *Eohadrotreta zhenbaensis* Zhang et al., Figures 3–6 in [12].

2017 *Eohadrotreta zhenbaensis* Zhang et al., Figures 2–7 in [12].

2018 *Eohadrotreta zhenbaensis* Zhang et al., Figures 1–3 in [8].

**Material.** Five specimens with conjoined valves, eight specimens with ventral valves, and twelve specimens with dorsal valves, all from the Guojiaba Formation.

**Diagnosis.** See Holmer and Popov in [41].

**Description.** The shell is ventribiconvex, subcircular to transversely oval in commissural outline (Figure 5I), on average 97% as wide as long (Table 2), with concentric growth lines on the shell surface. The ventral valve is low, conical to slightly convex, with a somewhat straight posterior margin bisected by a slightly recessed groove (Figure 5J). The ventral apex is moderately convex, situated on a rounded ventral larval valve slightly posterior to the posterior 1/3 of the shell length (Table 2); the ventral larval valve is nearly circular in outline, but perforated by the pedicle foramen, which is not enclosed by the larval shell (Figure 5K,L). The larval valve is ornamented by uniform flat-bottomed hemispherical pits approximately 1.03 μm in diameter. Concentric fila are present on the postlarval shell. The ventral pseudointerarea extends from apsacline to proline, and the interior is always poorly preserved. The dorsal valve is transversely oval in outline, on average 101% as long as wide (Table 2). The median septum is poorly developed. The dorsal cardinal muscle scars are prominent, occupying approximately 32% of the valve length and 38% of the valve width.

**Remarks.** See remarks for *E. zhujiahensis*.

**Occurrence.** The upper part of the Guojiaba Formation (lower Cambrian, Chiungchussuan Stage) of southern Shaanxi Province; the Shuijingtuo Formation of western Hubei Province; the middle Wulongqing Formation of eastern Yunnan, South China.

Genus *Kuangshanotreta* Zhang, Holmer and Hu, 2012

Type species. *Kuangshanotreta malungensis* Zhang, Holmer and Hu, 2012

*Kuangshanotreta malungensis* Zhang, Holmer and Hu, 2012

(Figure 5N–P)

2012 *Kuangshanotreta malungensis* Wang et al., Figures 2–4 in [43].

**Material.** Five specimens with ventral valves from the Guojiaba Formation.

**Diagnosis.** See Wang et al. [43].

**Description.** Shell subcircular in outline, about 93% as wide as long (Figure 5M, Table 3); outer surface of valves with pronounced growth lines (Figure 5N–P); ventral pseudointerarea apsacline; foramen posterior to umbo and moderately developed; dorsal pseudointerarea narrow, occupying about half of valve width; ventral visceral area circular, not extending to mid-valve; dorsal mid-septum extending anteriorly, slightly exceeding mid-valve; pedicle without visible central cavity, thread-like, protruding from post-umbonal foramen.

**Remarks.** *Kuangshanotreta malungensis* has been reported from the lower Cambrian Chengjiang fauna of South China [43]; shell range in diameter from 0.8 mm to 2.2 mm. Specimens are relatively small, about 0.5 mm–0.9 mm. Obvious concentric growth lines are preserved on the shell surface of both valves (Figure 5N–P). The shell outline and shape of *Kuangshanotreta* are somewhat similar to *Eohadrotreta* [24], but *Kuangshanotreta* has a less developed buttress, only has a weak dorsal median septum, and is generally larger than *Eohadrotreta* [43]. Nevertheless, it can be presumed that *Kuangshanotreta malungensis* specimens previously found manually from eastern Yunnan are equivalent to acid-etched specimens of *Eohadrotreta zhujiahenis* from the carbonates in western Hubei and southern Shaanxi provinces of South China [43]. Comparative studies and geometric analysis of specimens from these two genera have yet to be undertaken. 

**Occurrence.** The upper part of the Guojiaba Formation (lower Cambrian, Chiungchussuan Stage) of southern Shaanxi; the middle–upper part of the Heilinpu Formation of east Yunnan, South China.

Order Lingulida Waagen, 1885

Superfamily Linguloidea Menke, 1828

Family Obolidae King, 1846

Subfamily Obolidae King, 1846

Genus *Kyrshabaktella* Koneva, 1986

Type species: *Kyrshabaktella certa* Koneva, 1986

*Kyrshabaktella* sp.

(Figure 6A,B)

**Material.** Two specimens with dorsal valves from the Guojiaba Formation.

**Diagnosis.** See Skovsted et al. in [44].

**Figure 6:**
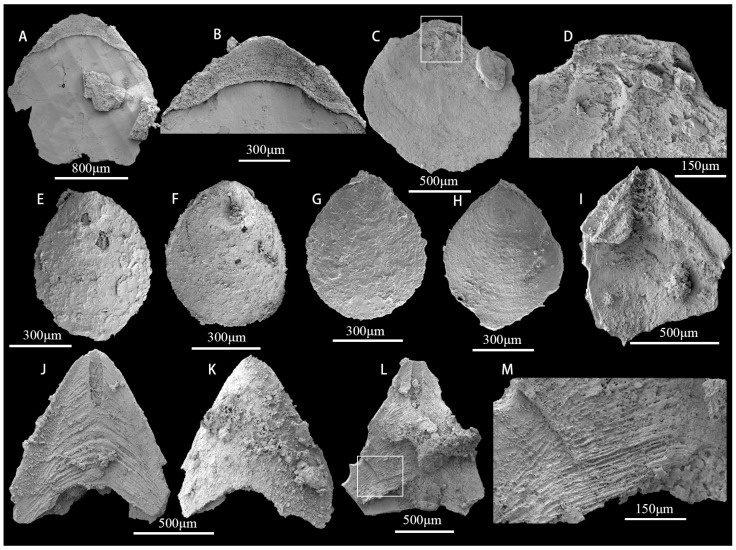
The Lingulida brachiopods from the upper Guojiaba Formation of the Dayingcun Section. (**A**) *Kyrshabaktella* sp., ELI–DYC–GJB+1–24, dorsal valve interior with pseudointerarea (**B**); (**C**) *Spinobolus* sp., ELI–DYC–GJB+1–22, dorsal valve interior and enlarged views of dorsal interior (**D**); (**E**–**H**) *Eoobolus* sp., ELI–DYC–GJB+2–8, ELI–DYC–GJB+0–3, ELI–DYC–GJB+0–86, ELI–DYC–GJB+0–88, internal mold; (**I**) *Eoobolus incipiens*, ELI–DYC–GJB+0–111, ventral valve interior with pseudointerarea. (**J**) *Lingulellotreta yuanshanensis*, ELI–DYC–GJB+1–14, details of a ventral pseudointerarea with well-defined flexure lines and propareas (**K**), bisected by a subparallel-sided pedicle groove (**J**); (**L**) *Lingulellotreta yuanshanensis*, ELI–DYC–GJB+2–1, ventral valve interior (**L**) and exterior molds (**M**).

**Description.** Valves oval to circular in outline (Figure 6A); weakly biconvex, slightly longer than wide, and have a maximum width anterior of mid-length. Dorsal valves are 1.6–1.9 mm long (mean 1.75, N = 2) and 1.6–1.8 mm wide (mean 1.7, N = 2). The ventral valve is gently convex, acuminate posterior, and rounded anteriorly. The dorsal valve is gently convex and oval in outline. The metamorphic shell is almost circular and smooth. The dorsal pseudointerarea is moderately raised above the valve floor occupying about 63% of the valve width (Figure 6B). The median groove is shallow broad, and weakly defined.

**Remarks.** On the basis of the outline of the ventral pseudointerarea with narrow propareas, a pedicle groove developing into an emarginature, and the low dorsal posterior pseudointerarea with broad margins, the species described above is referred to *Kyrshabaktella* [44,45]. *Kyrshabaktella* is assigned to the Family Zhanatellidae [45] as it lacks pitted larval and post-larval shells and possesses a smooth inner surface without special ornamentation. *Kyrshabaktella mudedirri* Kruse 1990, however, from the Middle Cambrian of Australia, has been previously shown to possess a badly preserved columnar shell structure [46]. Evidence of a well-preserved columnar shell structure is also evident in specimens from the lower Cambrian Harkless Formation of Nevada [44].

**Occurrence.** The upper part of the Guojiaba Formation (lower Cambrian, Chiungchussuan Stage) of South China. lower Cambrian of South Australia and Nevada, Amgan stage of Siberia.

Genus *Spinobolus* Zhang and Holmer, 2016

Type species: *Spinobolus popovi* Zhang and Holmer, 2016

*Spinobolus* sp.

(Figure 6C,D)

**Material.** A dorsal valve from the Guojiaba Formation.

**Diagnosis.** See Zhang et al. [7])

**Description.** The dorsal valve is thin and biconvex, subtriangular in outline (Figure 6C). The larval shell is smooth, with no ornamentation, pits, or pustules. The dorsal interior, with impressed posterolateral muscle scars, is bisected by an elongate median ridge that widens slightly and extends anteriorly along the median valve floor. The dorsal orthocline pseudointerarea is low and slightly elevated above the valve floor, separated by a broad, shallow median groove (Figure 6D). Posterolateral muscle scars of the dorsal valve interior are not well preserved. There are no mantle canal impressions in specimens from the upper Guojiaba Formation.

**Remarks.** *Spinobolus popovi* was described from the early Cambrian Shuijingtuo Formation in the Three Gorges region of South China with distinctive spinose ornamentation [7]. The same species of *Spinobolus* was also reported in the early Cambrian Xinji Formation in Ruicheng County of North China [35], which extended the geographical distribution of the genus. The genus was first discovered in the Shuijingtuo Formation without pitting of the metamorphic shell structure, but Zhang (2018) [8] later showed that it is present in the metamorphic shell of *Spinobolus*. In regards to the taxonomic position of this genus, we follow Zhang (2018) [8] in retaining the assignment of *Spinobolus* to the Obolidae King, 1846, as originally proposed [7].

**Occurrence.** The upper part of the Guojiaba Formation (lower Cambrian, Chiungchussuan Stage) of southern Shaanxi Province; Shuijingtuo Formation of western Hubei Province, South China.

Family Eoobolidae Holmer, Popov and Wrona, 1996

Genus *Eoobolus* Matthew, 1902

*Eoobolus* sp.

(Figure 6E–H)

**Material.** Four specimens with conjoined valves from the Guojiaba Formation.

**Description.** The ventral shell has a long oval shape and is slightly convex in outline (Figure 6E–H). The highest part of the shell is located in the mid-posterior part of the shell. The metamorphic shell structure is unclear. Detailed features could not be obtained as the shells are all preserved as internal molds.

**Remark.** *Eoobolus* has been described from the Shuijingtuo Formation in the Three Gorges area, often possesses a rugellae structure, and the shell structure consists mainly of secondary columnar shell layers [7]. *E.* aff. *viridis* from the Xihaoping Member of Dengying Formation in the southern Shaanxi area has a pustular structure, and the shell structure consists of secondary plate-like shell layers [9]. As the specimens from the Guojiaba Formation are all preserved in internal molds, it is not possible to assign these specimens to a particular species.

**Occurrence.** The upper part of the Guojiaba Formation (lower Cambrian, Chiungchussuan Stage) of southern Shaanxi Province; Shuijingtuo Formation of eastern Hubei Province, South China.

*Eoobolus incipiens* Zhang et al., 2021

(Figure 6I)

2001 *Eoobolus* aff. *viridis* Cobbold, Ushatinskaya and Holmer, p. 123, pl. 16, Figures 1–5, 10–13, 17 in [47].

2004 *Eoobolus* aff. *viridis* Li and Holmer, pp. 200–201, Figures 6 and 7 in [24].

2006 *Eoobolus* aff. *viridis* Jago et al., p. 414, Figure 4G in [48].

? 2016 *Eoobolus* aff. *viridis* Zhang et al., p. 347, Figure 10A–E in [7].

2016 *Eoobolus* sp. Betts et al., p. 194, Figure 14I–R in [32].

2018 *Eoobolus* aff. *viridis* Zhang, pp. 218–223, Figures 8.9–8.13 in [8].

2021 *Eoobolus incipiens* Zhang et al., p. 157, Figures 8–14 in [9].

**Material.** Three specimens with ventral valves from the Guojiaba Formation.

**Diagnosis.** See Holmer et al. [47]

**Description.** Shell slightly dorsiconvex, elongate subtriangular to suboval, approximately 117% as long as wide with rectimarginate anterior commissure (Figure 6I). The ventral valve is acuminate with an average apical angle of 98°, elongated subtriangular, generally convex, and approximately 117% longer than wide. The ventral valve is slightly convex in sagittal profile, with a maximum height slightly posterior to mid-length. Pseudointerarea orthocline, occupying 37% of valve length and 66% of valve width, with a deep, narrow, subtriangular pedicle groove, approximately 83% of pseudointerarea length, and 26% of pseudointerspace width. The proparea is broad, elevated above the valve floor, and divided into two almost equal parts by deep flexural lines. The ventral visceral area usually extends to about mid-length. The umbonal muscular scar is relatively well developed.

**Remark.** The ornamentation of *Eoobolus incipiens* includes pitting structures, prominent pustules, and drape structures [9]. Unfortunately, most metamorphic shells are generally exfoliated, and as a result, the preservation of these structures is poor [9]. Several occurrences of *Eoobolus* have been recorded in the early Cambrian strata of Australia [32,48,49,50]. Li and Holmer described a new species as *Eoobolus* aff. *viridis* Cobbold, 2001 [24]. *Eoobolus* from South China is considered conspecific with the specimens from the lower Parara Limestone of the Stansbury Basin and the lower Ajax Limestone and upper Wilkawillina Limestone in the Arrowie Basin of Australia [48]. *Eoobolus incipiens* differs from *E. malongensis* [51], as revised by Z. F. Zhang et al. [11], in that *E. malongensis* has a relatively smaller apical angle and a larger valve length–width ratio [11]. *Eoobolus*, due to its poorly preserved micro-ornamentation and similar shell outline and internal structure, can be difficult to distinguish from *Palaeobolus*, *Lingulella,* or *Ungula* [52]. *Eoobolus priscus*, found in Siberia, Antarctica, and Laurentia, differs from *E. incipiens* in having a smaller apical angle (80°–90°), a weekly developed dorsal median tongue and median ridge, a rudimentary umbonal musculature and slightly elongated valves [52,53,54]. Poulsen suggests that *Lingulella siniella* is a junior synonym of *Eoobolus priscus* because of its strong morphological similarity [55]. Thus, the valid species from the Siberian platform are *Eoobolus siniellus*, *E. variabilis*, *E. priscus*, and *E. pelmani* [52].

**Occurrence.** The upper part of the Guojiaba Formation (lower Cambrian, Chiungchussuan Stage) and Xihaoping Member of southern Shaanxi Province, South China; Australia.

Family Lingulellotretidae Koneva and Popov, 1983

Genus Lingulellotreta malongensis Koneva, 1983

Type species: *Lingulellotreta ergalievi* Koneva, 1983

*Lingulellotreta yuanshanensis* Zhang et al., 2020

(Figure 6J–M)

1993 *Lingulepis malongensis* Rong, Jin et al., p. 794, Figures 5.1, 5.6, 5.7, 8.1–8.4, 9.4 in [56].

1994 *Lingulepis malongensis* Rong, Luo, Jiang and Tang: pl. 37, Figures 11–14 in [57].

2000 *Lingulellotreta malongensis* Rong, Holmer and Popov: 72, Figure 34, 1a–d in [40].

2001 *Lingulellotreta malongensis* Rong, Holmer, Popov Koneva and Bassett: 56, pl. 13, Figures 11, 13–15 in [45]. (synonymy)

2004 *Lingulellotreta malongensis* Zhang et al., Figures 1–4 in [58].

2004 *Lingulellotreta malongensis* Hou et al., pp. 182–183, Figures 17.3 and 17.4 in [59].

?2004 *Lingulellotreta malongensis* Rong, Li and Holmer: 199, Figure 9 in [24]. (synonymy)

2005 *Lingulellotreta malongensis* Zhang et al., Figures 1–3 in [60].

2007 *Lingulellotreta malongensis* Zhang et al., Figures 1–3 in [61].

2008 *Lingulellotreta malongensis* Zhang et al., Figure 4K–N in [62].

?2015 *Lingulellotreta malongensis* Rong, Z. F. Zhang, Zhang, Holmer and Li: 176, Figure 5 in [63]. (synonymy)

?2016 *Lingulellotreta malongensis* Rong, Z. F. Zhang, Zhang, Li and Holmer: 348, Figure 10f in [7].

2020 *Lingulellotreta yuanshanensis* Zhang et al., p. 23, Figures 7 and 8 in [11].

**Material.** Five specimens with ventral valves from the Guojiaba Formation.

**Diagnosis.** See Zhang et al. [11].

**Description.** Shell subtriangular to oblong subovate in outline. The ventral valve is slightly convex, on average 138% longer than wide in adults (up to 1.62 mm long; Figure 6J–L; Table 4). The ventral pseudointerarea is elongated, triangular, about 56% longer in length, and occupying 88% of the total valve width in adults. Orthogonal pseudointerarea extends anteriorly to approximately 56% of the total valve length in adults (Figure 6J,L) with well-developed bend lines (Figure 6M). Elongated oval pedicle foramen located at the posterior tip of pseudointerarea with an average apical angle of 55.1°. Pedicle foramen up to 0. 48 mm long and 0.1 mm wide, up to about 26% of the shell length in adults.

**Remark.** *Lingulellotreta malongensis* is one of the most distinctive early Cambrian linguloids from Kazakhstan and South China. The shell size of specimens from Kazakhstan is about half that of those recorded from South China [56,64]. The species is the earliest known taxon of the family Lingulellotretidae, and according to a detailed description by Holmer et al. (1997) [64], it is mainly recognized by its elongated foramen, internal pedicle tube, and smooth larval shell. These species were also found in the Chengjiang fauna with the digestive canal in a U-shaped arrangement [58]. *Lingulellotreta ergalievi* Koneva, 1983, is considered a junior synonym of *Lingulellotreta malongensis* [50]. The specimens presented here are only about half the length of the Kazakhstan specimens and a quarter of the length of the Chengjiang specimens [24]. This means that, from the Chiungchussuan Stage to the Tsanglangpuian Stage, the length of the pseudointerarea of *Lingulellotreta* gradually decreases, with a corresponding extension anterior to the posterior wall [64]. The elongated pseudointerarea may be a factor leading to the extinction of this species in the middle Cambrian [64]. The Chengjiang specimens of *Lingulellotreta yuanshanensis* were erected as a new species based on comparison to the type species *Lingulellotreta ergalievi. Lingulellotreta yuanshanensis*, when compared to *L. ergalievi*, has a more elongated ventral pseudointerarea, accommodating an extended body cavity, deepening posteriorly, with a recurved digestive tract [11].

**Occurrence.** The upper part of the Guojiaba Formation (lower Cambrian, Chiungchussuan Stage) in the Dayingcun Section, Shuijingtuo Formation in the Xiaoyangba Section of southern Shaanxi Province; Shuijingtuo Formation in the Aijiahe Section of Hubei Province, South China; Kazakhstan.

Phylum Cnidaria Hatscheck, 1888

Class, order, and family uncertain

Genus *Sphenothallus* Hall, 1847

Type species: *Sphenothallus angustifolius* Hall, 1847

*Sphenothallus* sp.

(Figure 7)

**Material.** Approximately 187 specimens from the Guojiaba Formation, the proximal part of the holdfast was not preserved.

**Figure 7:**
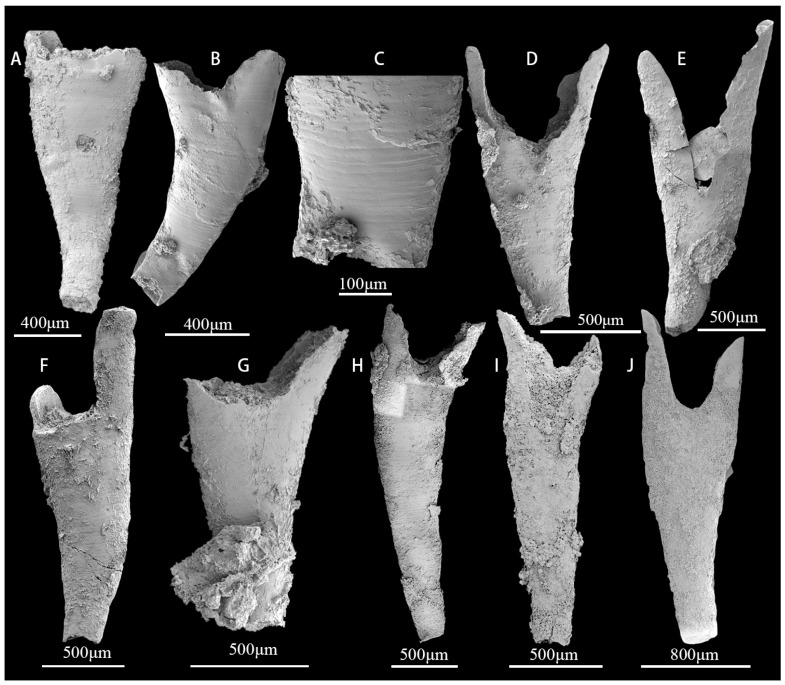
*Sphenothallus* sp. from the upper Guojiaba Formation of the Dayingcun Section. (**A**) ELI–DYC–GJB+0–104, internal mold with biological drilling; (**B**,**C**) ELI–DYC–GJB+0–6, curved sample (**B**) with transverse striation (**C**); (**D**) ELI–DYC–GJB+0–97, showing a V-shaped notch; (**E**) ELI–DYC–GJB+1–8, showing U-shaped notch; (**F**) ELI–DYC–GJB+1–33, uncomplete sample with transverse striation; (**G**) ELI–DYC–GJB+1–7, the broken notch; (**H**) ELI–DYC–GJB+2–5, slender sample; (**I**) ELI–DYC–GJB+4–25, internal mold specimen; (**J**) ELI–DYC–GJB+4–17, internal mold with U-shaped notch.

**Description.** Each specimen is solitary and incomplete without a holdfast. Some specimens have circular holes (Figure 7A). All specimens are composed mainly of calcium phosphate and organic material. The length of the specimens is between 1 mm and 3 mm (Figure 7A–J), gradually tapered, open at the apical end, usually with the fracture edge at the apical end of each face forming a V-shaped (Figure 7E) or U-shaped (Figure 7D,J) notch. The inner surface is filled, and the outer surface is smooth or with numerous (Figure 7C) transverse striae, straight or gently curved towards the opening.

**Remarks.** *Sphenothallus* Hall, 1847 occurs in Palaeozoic marine strata [25,65,66,67,68,69]. Phosphatic examples of *Sphenothallus* fossils have received less attention over the last century than *Sphenothallus* from shale facies. Lower Cambrian examples of *Sphenothallus* from the Guojiaba and Xiannvdong Formations in the Shaanxi region were previously reported by Li et al. (2004b) [25]. *Sphenothallus songlinensis* from the Niutitang Formation and a potential *Sphenothallus taijiangensis* species from the Kaili Formation have both been described from Guizhou Province [65,70,71]. These previous specimens are all preserved in two dimensions, whereas the fossils from the Guojiaba Formation are preserved in three dimensions. Other unidentified *Sphenothallus* species have been found in the lower Middle Cambrian Burgess Shale Formation of British Columbia [72]. Some *Sphenothallus* species are from the Late Ordovician and are found in the United States and Baltica. These specimens have revealed the mineral composition and microstructure of phosphatic *Sphenothallus* species [72,73]. The characteristics and stratigraphic range of phosphatic *Sphenothallus* require further study.

**Occurrence.** The upper part of the Guojiaba and all Xiannvdong Formations (lower Cambrian, Chiungchussuan, and early Tsanglangpuian stages) of southern Shaanxi Province; Niutitang Formation and Kaili Formation, Guizhou Province, South China; the Burgess Shale Formation, British Columbia; the United States, Baltica, Brazil, Korea.

Phylum Porifera Grant, 1826

Class Archaeocyatha Bornemann, 1844

Order Ajacicyarthida R.Bedford and J.Bedford, 1939

Suborder Ajacicyarthina R.Bedford and J.Bedford, 1939

Superfamily Bronchocyathoidea R.Bedford and J.Bedford, 1939

Family Ajacicyathidae R.Bedford and J.Bedford, 1939

Genus *Robustocyathellus* Konyushkov, 1972

*Robustocyathellus* sp.

(Figure 8A–H)

**Material.** Approximately 72 specimens, 12 complete specimens, and 60 broken specimens from the Guojiaba Formation.

**Figure 8:**
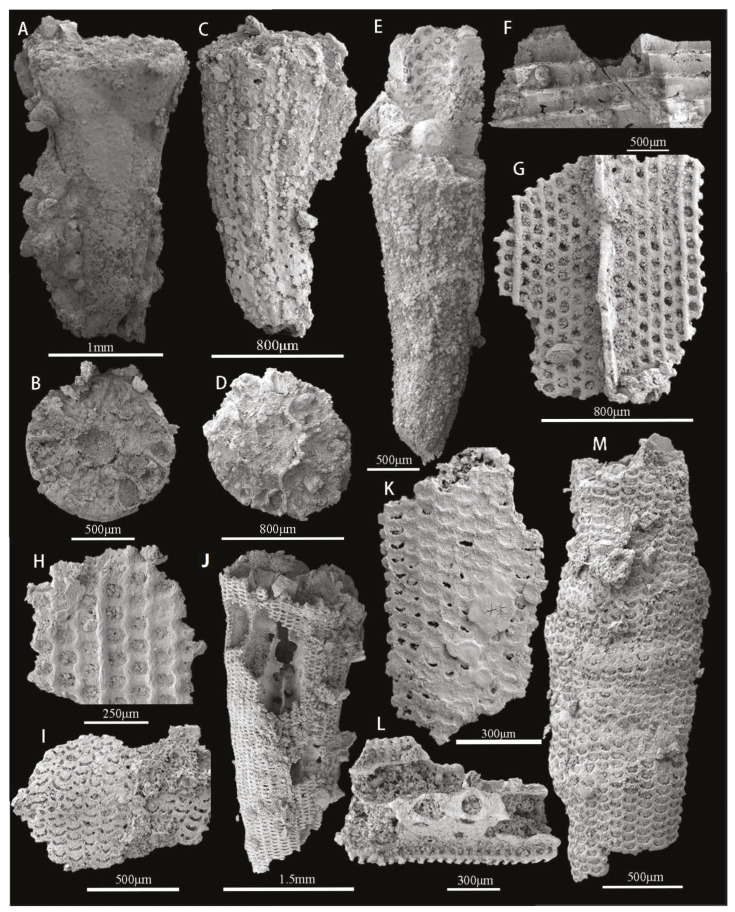
The archaeocyathans *Robustocyathellus* sp. (**A**–**H**) and *Yukonocyathus* sp. (**I**–**M**) from sample sites GJB+0 and GJB+1 of the upper Guojiaba Formation of the Dayingcun Section. (**A**,**B**) ELI–DYC–GJB+1–25, complete specimen and vertical view; (**C**,**D**) ELI–DYC–GJB+1–4, complete specimen and vertical view; (**E**) ELI–DYC–GJB+0–13, complete specimen with pores of the inner wall; (**F**) ELI–DYC–GJB+1–6, broken septa; (**G**) ELI–DYC–GJB+0–16, 6-7 rows pores in the outer wall per intercept; (**H**) ELI–DYC–GJB+0–91, incomplete outer wall with normal pores; (**I**) ELI–DYC–GJB+1–19, broken outer wall and slitlike pores; (**G**) ELI–DYC–GJB+0–96, complete specimen and no tabula; (**K**,**L**) ELI–DYC–GJB+1–39, (**K**) outer wall with simple pores, (**L**) left view of I1, bearing supplementary bracts externally on the outer wall; (**M**) ELI–DYC–GJB+1–8, outer wall with slitlike pores.

**Description.** Cups are cylindrical or conical (Figure 8A,C,E), with a minimum length of 1.83 mm (Figure 8C) and a maximum of 5.91 mm (Figure 8E). Diameter up to 1 mm or more (Figure 8B,D). The outer wall with simple pores is arranged longitudinally (Figure 8G,H), with additional bracts on the outside of the outer wall. The diameter of the pore is between 45.5 and 86.5 μm. The inner wall is thicker, 0.09–1.1 mm, with one row of large and round pores per intercept; the diameter of the pores is between 55.7 and 66.3 μm. No pores or only one row of pores in the septa (Figure 8F). The cup cone angle is 22–29 degrees, but due to the variability of preservation, there is some margin of error in the measurements.

**Remarks.** *Robustocyathus* also occurs in South China, Siberia, eastern and western Gondwana, and western Laurentia [74]. Based on the identified fossil assemblages of archaeocyaths in Siberia, three zones are established [75]. *Robustocyathus legitimus* was found in the lower part of the Sekten Formation of the Siberian Platform [76]. *Robustocyathus* has also been found in the lower Cambrian reef facies of the middle Lena River [77]. In Nevada, USA, the archaeocyath species of Gold Point Reef are low in diversity in the lower Poleta Formation [78]. *Robustocyathus infundibulus* was discovered in the Qiongzhusian Stage of South China [74]. The identification of *Robustocyathus* [79], *Usloncyathus* [79], *Stillicidocyathus* [80], *Metacyathellus* [80], *Dictyocyathus* [80], and *Agastrocyathus* [81] is significant. Due to the restricted stratigraphic range of these taxa, direct correlations with Siberia, Eastern and Western Gondwana, and Laurentia are possible at a higher resolution level [28,74].

**Occurrence.** The upper part of the Guojiaba and Xiannvdong Formations (lower Cambrian, Chiungchussuan, and early Tsanglangpuian stages) of southern Shaanxi Province; Shuijingtuo Formation, Hubei Province, South China; Tommotian Stage, Siberia.

Superfamily Ethmophylloidea Okulitch, 1937

Family Fallocyathidae Rozanov, 1969

Genus *Yukonocyathus* Handfield, 1971

*Yukonocyathus* sp.

(Figure 8I–M)

**Material.** Approximately 16 specimens from the Guojiaba Formation.

**Description.** Cups are columnar (Figure 8G), and the length of the cup is 1.31–3.69 mm in the longitudinal direction. Diameter up to 1.68 mm. Outer wall with horizontal to upwardly projecting S-shaped canals (Figure 8I,G,K,M), bearing additional bracts on the outside (Figure 8L). Inner wall with one row of simple pores per intercept (Figure 8G), formed by fluting of the inner edges of the septa; septa aporose to sparsely porous.

**Remarks.** The literature on *Yukonocyathus* is sparse. In the United States, the majority of irregular archaeocyath taxa belonging to the order Syringocnemidida and regular archaeocyaths are assigned to the family Ethmophyllidae [82]. The most abundant genera in the USA are *Sekwicyathus*, *Yukonocyathus*, *Cordilleracyathus*, *Ethmophyllum,* and *Pesudosytingocnema* [82]. In Sardinia, the genus *Yukonocyathus* is characterized by the presence of an outer wall with S-shaped canals and a simple inner wall [77], which are consistent with the specimens described here.

**Occurrence.** Upper part of the Guojiaba Formations (lower Cambrian, Chiungchussuan, and early Tanglangpuan stages) of southern Shaanxi Province, South China; Canada, United States.

## 6. Regional Fossil Occurrences and Stratigraphic Correlations of the Guojiaba Formation

**Figure 9:**
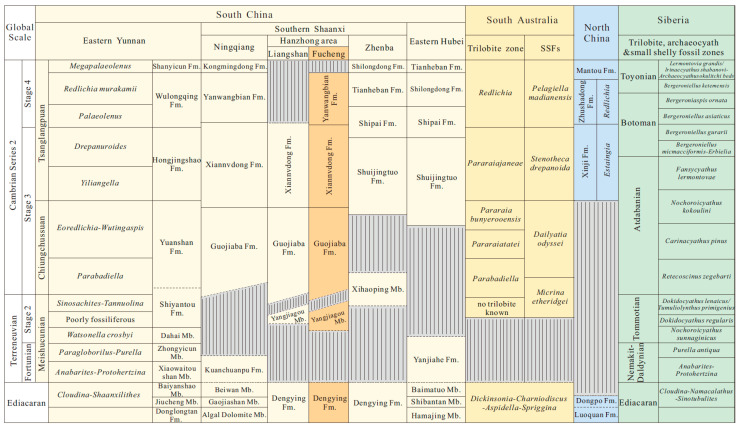
Preliminary correlation diagram for Cambrian stages 3–4 and trilobite zones of South China, East Gondwana, North China, and Siberia. The bright yellow indicates the main research stratum. Modified from Zhang ZF et al., 2016, Zhang ZL et al., 2021 [7,9].

The presence of three-dimensional preserved archaeocyathan fossils among the SSFs mentioned above is undoubtedly evident. There are relatively few reports of phosphatized archaeocyathan fossils [83,84]. Archaeocyath species were the earliest metazoan reef builders. They had calcareous skeletons and lived on the sea floor. The archaeocyathans flourished briefly in the early Cambrian. The fossils were almost ubiquitous in tropical regions during most of the early Cambrian [85]. For example, they were common in East Gondwana (Australia, East Antarctica), West Gondwana (Spain, France), and both coasts of Laurentia (eastern Canada, Nevada) [86,87,88,89,90]. Archaeocyaths first came to prominence in the Tommotian Stage of Siberia before the trilobites. The archaeocyaths were associated with SSFs. They are found together with chancelloriids, calcareous sponges, stromatoporoids, hyoliths, trilobites, lingulid brachiopods, and assorted echinoderms [85,87,91,92]. Archaeocyaths were first reported from the Three Gorges area of China [93]. The archaeocyathan fossils were successively collected in the Shuijingtuo Formation, Xiannvdong Formation, Tianheban Formation, Mingxinsi Formation, etc., of South China [28]. The oldest archaeocyaths in China are found in the Xiannvdong Formation of southern Shaanxi [74]. However, the discovery of phosphatized archaeocyathans from the Guojiaba Formation shows that the age of appearance of archaeocyaths extends to the Late Chiungchussuan Stage, which is the first report in southern Shaanxi of China. Yuan et al. (2001) proposed the evolutionary sequence of three Cambrian archaeocyathans assemblages, including the Chiungchussuan archaeocyathan assemblage, the Tsanglangpuian archaeocyathan assemblage, and the Tienhopan archaeocyathan assemblage [28]. These three archaeocyathans assemblages may be useful for the international correlation of lower Cambrian strata [28,94]. The Chiungchussuan archaeocyathan assemblage is similar to the archaeocyathan assemblage of the Tommotian to Botoman in Siberia, the Tsanglangpuian archaeocyathan assemblage is similar to the lower Toyonian Stage, and the Tienhopan archaeocyathan assemblage is similar to the upper Toyonian Stage (Figure 9) [28,94].

Hyolithelminths, chancelloriids, echinoderm plates, bradoriids, oocysts, etc., have not been described from the Guojiaba Formation in previous studies. The SSFs described in this study markedly increase the overall diversity known from Stage 3 Cambrian Series 2 in the Fucheng area. Trilobites *Wutingaspis–Eoredlichia* Zone has been discovered in the Guojiaba Formation, the lower Shuijingtuo Formation of the Hubei area, and the Yu’anshan Formation of the Yunnan area [95]. Most of these trilobites belong to the genus *Eoredlichia* but are poorly preserved [96]. The *Wutingaspis–Eoredlichia* Zone is widely found in various regions of the world, including South China, Siberia, Australia, Italy, and Morocco, suggesting a potential global correlation [95]. Two trilobite biozones have been identified in the Guojiaba Formation [97]. The lower part of the Guojiaba Formation is dominated by *Parabadiella* and *Tsunyidiscus*, the upper part by *Eoredlichia* and *Wutingaspis*, and other assemblages contain brachiopods and other fossils, suggesting that it could be correlated to the Yu’anshan Formation of Yunnan in terms of fossil assemblages [98]. 

Traditionally, the Yu’anshan Formation in eastern Yunnan is correlated with the Shuijingtuo Formation in southeastern Shaanxi and western Hubei provinces, both because the two formations represent the regional lowermost trilobite-bearing horizons respectively, and partly because they were deposited in the same sedimentary environment characterized by a sequence of black shales in the base of the two formations. As discussed by Zhang et al. (2016) [7], the multi-taxa trilobites recovered in the Yu’anshan and Shuijingtuo formations are most likely diachronous, and thus other fossils, notably SSFs dissolved from the carbonate interbeds, are of stratigraphical importance in the intra- and interregional stratigraphic correlation. More importantly, the Xihaoping Member of Dengying Formation, disconformably underlying the Shuijingtuo Formation in the Zhenba area, was recently revealed to yield the trilobite *Parabadiella huoi*, but no *Eoredlichia* found [9]. In terms of yielding the same trilobite species of *Parabadiella*, it is therefore assumed that the Xihaoping Member of Dengying Formation is well-correlated with the lower Guojiaba Formation, which contains rich exoskeletons of *Parabadiella* (Figure 9). Such stratigraphic correlations across the regions of South China have been well supported by the discovery of SSFs in these regions.

The Xihaoping Member of Dengying Formation in the Zhenba area contains the trilobite *Parabadiella huoi*, but no *Eoredlichia* has been found [9]. The fossil assemblages of the Xihaoping Member of Dengying Formation consist mainly of *Eoobolus incipiens* without the *Eohadrotreta zhenbaensis*, *E. zhujiahensis*, and *Lingulellotreta yuanshanensis*. It should, therefore, likely be assigned to early Stage 3 of Cambrian Series 2 (Figure 9) [9]. The fossil assemblage of the Shuijingtuo Formation is dominated by *Eohadrotreta zhenbaensis* and contains other taxa similar to those of the Hongjingshao Formation and Guanshan Fauna in eastern Yunnan. This means the Shuijingtuo Formation is relatively younger than the Yu’anshan Formation (Figure 9) [9,99,100]. The fossil assemblage of the *Dailyatia obyssei* Zone from Australia contains *Parabadiella* and other skeletal fossils [60]. The age of the *Dailyatia obyssei* Zone can be considered comparable to that of the Guojiaba Formation. Based on the assemblage found in the Guojiaba Formation, it could be concluded that the Guojiaba Formation should belong to Stage 3 of Cambrian Series 2.

**Figure 10:**
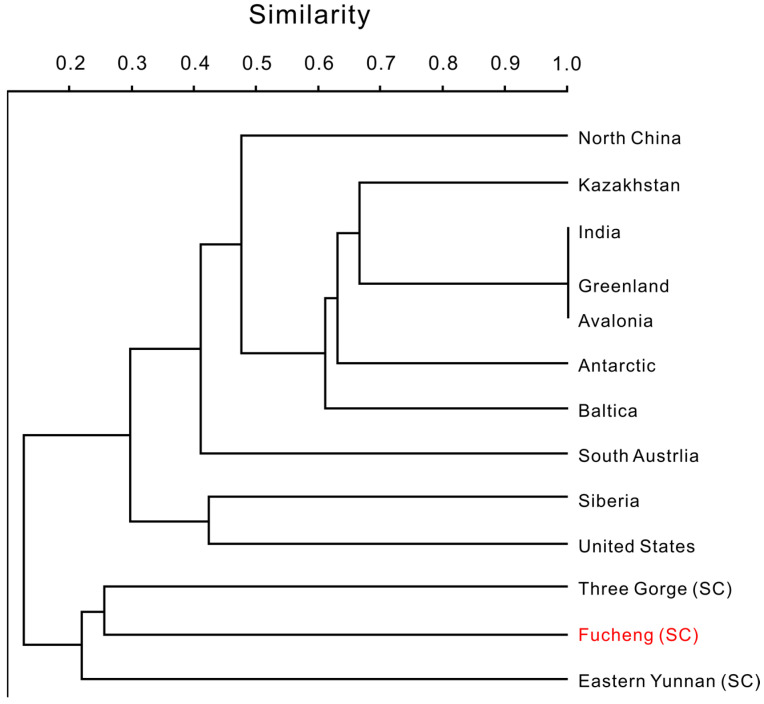
Results of the pair-group cluster analysis (Jaccard similarity) based on SSF alpha diversity in the Dayingcun Section and other lower Cambrian faunas. The Guojiaba Formation is most similar to eastern Yunnan Province and the Three Gorges of Hubei Province in China.

Cluster analysis shows that the lower Cambrian SSF assemblage from the Dayingcun Section together with the eastern Yunnan Province and Three Gorges of China, although the similarity between the three faunas is still marginal (Figure 10). What similarity exists is due to the co-occurrence of the genera *Eohadrotreta*, *Kuangshanotreta*, *Kyrshabaktella*, *Spinobolus*, *Lingulellotreta*, *Eoobolus*, *Sphenothallus*, *Kunmingella*, and *Robustocyathellus*. The presence of acrotretoids indicates that the Guojiaba Formation can be correlated with the upper silty shales of the Yu’anshan Formation in Yunnan Province (Figure 9) [7,11,43,101]. The acrotretid *Eohadrotreta zhenbaensis* was obtained from the Shuijingtuo Formation of the Yangtze Platform [9,22,24], the Xinji Formation of the North China Platform [35], the Tethyan Himalaya [102], the *Dailyatia dyssey* Zone of South Australia [60], and the uppermost Shackleton Limestone [103] (Figure 10). The acrotretid *Eohadrotreta zhujiahensis* occurs in the Shuijingtuo, Xiannvdong, and Guojiaba Formations of South China [24,104]. The acrotretid *Kuangshanotreta malungensis* is recovered from the Yu’anshan Formation of South China. The lingulid *Spinobolus* extends the range from South China to North China [7,35].* Lingulellotreta yuanshanensis* is reported in this study for the first time from the upper Guojiaba Formation, and specimens appear to be similar to the *Lingulellotreta yuanshanensis* from the silty shales of the Yu’anshan Formation in the Chengjiang area, with both possessing an elongated ventral pseudointerarea [11]. The lingulid *Lingulellotreta yuanshanensis* is restricted to South China [11], with limitations and territoriality. The biostratigraphy correlates closely with the brachiopods recently reported from the Yu’anshan Formation in the Yunnan area, as well as brachiopods found from the *Dailyatia obyssei* Zone of Australia (Figure 10) [103]. This suggests the possibility of an association between these contemporary brachiopod faunas. Sphenothallids from the Guojiaba Formation are more abundant than those from the Chengjiang Formation, likely because the Guojiaba Formation represents a relatively shallower environment [105]. The current affinity for *Sphenothallus* is thought to be related to cnidarians [106]. *Sphenothallus* was widespread in Baltica [72], Laurentia [66,107], South China [25,65], and Siberia [104] and was considered a marine invertebrate [108]. *Kunmingella* *douvillei* has been previously found in the Shaanxi and Sichuan regions of the South China platform [39,97], with large quantities in the Yu’anshan Formation (Figure 10) [109]. Chancelloriids of *Chancelloria* sp., *Allonnia* sp., and *Archiasterella* sp. have been discovered from the early Cambrian and widely distributed in South China [15], the *Dokidocyathus regularis* Zone of the Tommotian Stage in Siberia [84], the Laurentia [31,104], and North China [15]. The archaeocyathans of *Robustocyathellus* sp. are found in the Guojiaba Formation and Xiannvdong Formation of the Fucheng area, southern Shaanxi. Many of fossils discovered in the Guojiaba Formation have regional distribution characteristics, bearing little similarity with South Australia, the Baltic, Siberia, Avalon, North China, and other regions (Figure 10). Given the links between the Guojiaba Formation taxa described here and those from other regions, the hyolithelminths, brachiopods, sphenothallids, archaeocyathans, chancelloriids, echinoderms, trilobites, and bradoriids reported here have the potential to expand the ability to correlate the lower Cambrian strata of South China with other strata both regionally and internationally.

## 7. Conclusions

The SSF assemblage of the upper Guojiaba Formation is reported for the first time. The earliest archaeocyathan, brachiopods of Acrotretida *Eohadrotreta zhujiahensis* and *E. zhenbaensis* and Lingulida *Lingulellotreta yuanshanensis* are discovered in this area. It also revealing the benthic diversity of the early Cambrian fauna dominated by *Sphenothallus* sp. This provides new fossil evidence for the early Cambrian stratigraphic correlation in the Micang Mountain area of southern Shaanxi.

## Figures and Tables

**Table 1:** Ventral valve measurements of *Eohadrotreta zhujiahensis* from the Guojiaba Formation.

V	L	W	Lms	Wms	Lf	Wf	L/W	Lms/L	Lf/L	Lf/Wf
Count	13	18	17	17	14	15	12	12	8	14
Min	478	485	99	127	31	51	0.84	0.15	0.06	0.4
Max	907	925	195	223	76	99	1.09	0.30	0.14	1.06
Mean	632	625	138	177	60	82	0.95	0.22	0.09	0.4
SD	110	125	32	32	12	15	0.08	0.04	0.026	0.15

V, ventral valve. L, W, length and width. Lms, Ws, length and width of metamorphic shell. Lf, Wf, length and width of pedicle foramen. All measurements are in micrometers.

**Table 2:** Ventral valve measurements of *Eohadrotreta zhenbaensis* from the Guojiaba Formation.

**V**	**L**	**W**	**H**	**Lms**	**Wms**	**Lf**	**Wf**	**L/W**	**Lms/L**	**Lf/L**
Count	8	8	5	5	4	4	8	8	4	4
Min	544	580	262	174	192	59	57	0.81	0.29	0.07
Max	1034	1265	394	204	226	91	84	1.12	0.30	0.14
Mean	734	760	328	182	208	79	74	0.97	0.30	0.11
SD	177	227	62	17	13	9	10	0.11	0.003	0.022
**D**	**L**	**W**	**Lms**	**Wms**	**Lp**	**Wp**	**Lc**	**Wc**	**L/W**	**Lms/L**
Count	12	12	11	11	2	2	2	2	12	11
Min	648	608	180	216	305	76	228	323	0.74	0.22
Max	1360	1316	426	419	383	114	434	430	1.24	0.43
Mean	893	889	294	343	344	95	331	376	1.01	0.32
SD	225	227	81	77	55	27	145	75	0.12	0.06

V, ventral valve and D, dorsal valve. L, W, length and width. H, height. Lms, Wms, length and width of metamorphic shell. Lp, Wp, length and width of dorsal valves pseudointerarea. Lf, Wf, length and width of pedicle foramen. Lc, Wc, length and width of cardinal muscle scar. All measurements are in micrometers.

**Table 3:** Ventral valve measurements of *Kuangshanotreta malungensis* from the Guojiaba Formation.

V	L	W	Lms	Wms	Lf	Wf	L/W	Lms/L	Lf/L
Count	5	5	5	5	2	2	5	5	2
Min	588	627	180	213	31	39	0.87	0.23	0.05
Max	988	947	236	260	65	47	1.04	0.30	0.06
Mean	771	824	209	236	48	43	0.93	0.27	0.05
SD	143	119	23	20	23	5	0.06	0.03	0.01

V, ventral valve. L, length; W, width. Lms, Wms, length and width of metamorphic shell. Lf, Wf, length and width of pedicle foramen. All measurements are in micrometers.

**Table 4:** Ventral valve measurements of *Lingulellotreta yuanshanensis* from the Guojiaba Formation.

V	L	W	Il	Iw	Pgl	Pgw	Aa	L/W	Il/Iw	Il/L	Pgl/L
Count	5	5	5	5	5	5	5	5	5	5	5
Min	1378	986	820	884	350	88	52	1.25	0.84	0.53	0.23
Max	1623	1199	880	1039	485	103	57	1.5	0.92	0.59	0.29
Mean	1503	1088	850	959	394	95	55	1.38	0.88	0.56	0.26
SD	94	86	24	61	54	5	2	0.08	0.03	0.02	0.02

V, ventral valve. L, length; W, width. Il, Iw, length and width of ventral valves pseudointerarea. Pg, length of pedicle groove. Aa, apical angle. All measurements are in micrometers.

## Data Availability

Not applicable.
